# Bladder metastasis from lung adenocarcinoma: a difficult differential diagnosis with primary bladder adenocarcinoma

**DOI:** 10.1186/1477-7819-12-90

**Published:** 2014-04-09

**Authors:** Luigi Cormio, Francesca Sanguedolce, Giuseppe Di Fino, Paolo Massenio, Giuseppe Liuzzi, Pantaleo Bufo, Giuseppe Carrieri

**Affiliations:** 1 Department of Urology and Renal Transplantation and Department of Pathology, University of Foggia, Viale L. Pinto 1, 71121 Foggia, Italy

**Keywords:** Urinary bladder, Lung cancer, Adenocarcinoma, Metastasis

## Abstract

**Background:**

Bladder metastases from lung adenocarcinoma are extremely rare; in the seven previously reported cases, the finding of an intact epithelium overlying the bladder tumour was considered suggestive of a secondary lesion. We describe the first case of bladder metastasis from lung adenocarcinoma whereby endoscopic appearance was strongly consistent with primary bladder cancer, thus complicating the differential diagnosis with primary bladder adenocarcinoma.

**Case report:**

A 65-year-old woman with a 13-year history of clean intermittent catheterization was diagnosed with a right lung adenocarcinoma metastatic to mediastinal and right supraclavicular nodes, as well as to the left lung, and treated with six cycles of cisplatin/pemetrexed, followed by six cycles of pemetrexed only. The 18-month follow-up computed tomography revealed several solid lesions of the bladder wall and she was scheduled for transurethral resection of bladder tumours. Endoscopic appearance was strongly consistent with primary bladder cancer but a thorough pathologic evaluation allowed the diagnosis of bladder metastasis from lung adenocarcinoma.

**Conclusions:**

Differentiating primary bladder adenocarcinoma from metastatic adenocarcinoma lesions can be difficult. An endoscopic appearance consistent with primary bladder cancer further complicates the differential diagnosis, which heavily relies on pathologic evaluation and specific immunohistochemical staining.

## Background

Lung cancer is the leading cause of cancer-related death worldwide and adenocarcinoma is the most common cell type, representing approximately 50% of all lung cancer cases [1]. Bladder metastases from lung cancer are uncommon; those originating from lung adenocarcinoma are extremely rare. Reviewing the literature, we found only seven documented cases of bladder metastasis from lung adenocarcinoma, all pointing out difficulties in differentiating secondary adenocarcinoma lesions from primary bladder adenocarcinoma [2-6]. ln all previously reported cases, the finding of an intact epithelium overlying the bladder tumour was considered suggestive of a secondary lesion, thus contributing to the differential diagnosis [3]. Herein, we describe the first case of bladder metastasis from lung adenocarcinoma in which the endoscopic appearance was strongly consistent with primary bladder cancer, and discuss the diagnostic and therapeutic challenges of such uncommon clinical condition.

## Case presentation

**Figure 1:**
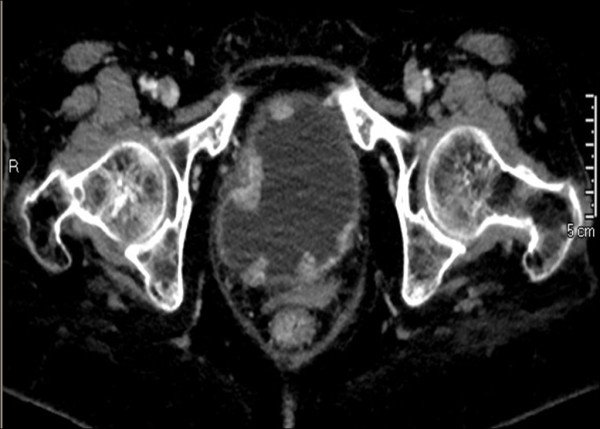
**Abdominal computed tomography.** Several solid lesions protruding into the bladder lumen are seen.

**Figure 2:**
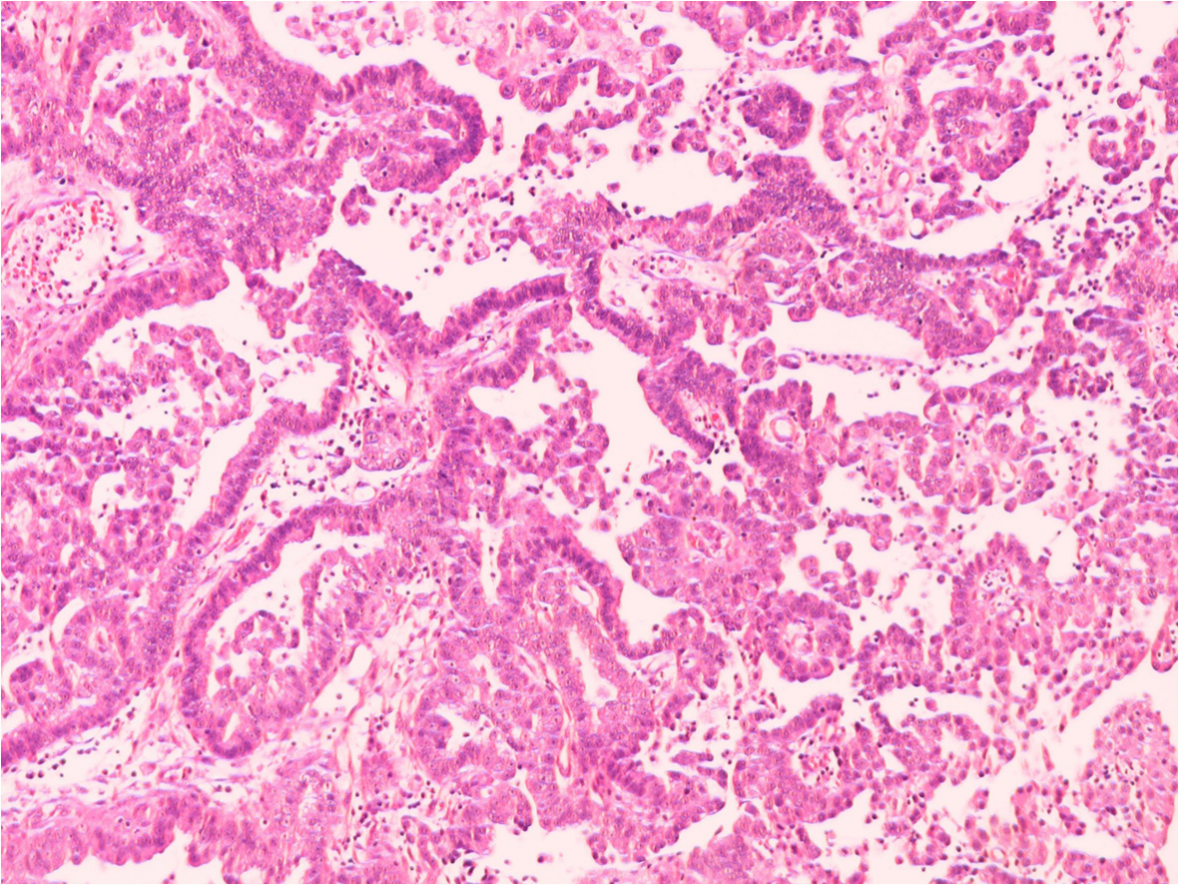
**Lung adenocarcinoma metastatic to the bladder.** Tumour cells with high nuclear atypia are arranged in alveolar (not shown) and papillary pattern (hematoxylin & eosin, original magnification x200).

**Figure 3:**
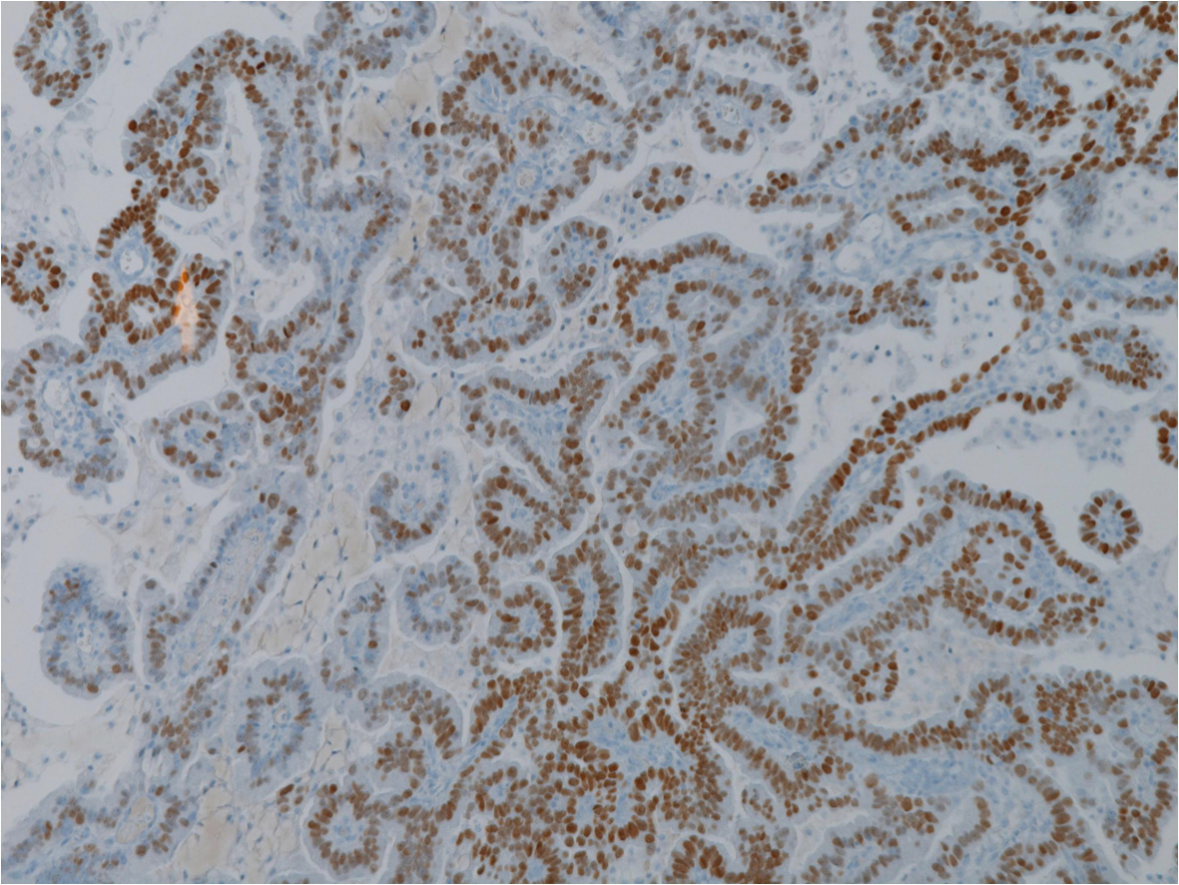
Tumour cells show nuclear staining for TTF-1 (original magnification x200).

A 65-year-old Caucasian woman with a 20 cigarette/day smoking history was diagnosed in November 2011 with a right lung adenocarcinoma metastatic to mediastinal and right supraclavicular nodes as well as to the left lung, stage T2N3M1b. She was given six cycles of chemotherapy with cisplatin and pemetrexed, and then maintenance chemotherapy with six cycles of pemetrexed only. Follow-up computed tomography (CT) scans, performed every six months, showed stable disease until February 2013, when several solid lesions protruding into the bladder lumen were revealed (Figure 1). The patient had never had gross hematuria although she had been on clean intermittent catheterization since 2000 because of a car accident resulting in a severely hypotonic bladder. She was scheduled for transurethral resection of the bladder tumours; their endoscopic appearance was strongly consistent with primary bladder cancer. Pathology showed tumour cells arranged in alveolar and papillary pattern deeply infiltrating the bladder wall (Figure 2). Immunohistochemical staining was positive for thyroid transcription factor 1 (TTF-1) (Figure 3), cytokeratin 7 (CK7) and mucin 1 cell surface associated (MUC1), while negative for cytokeratin 20 (CK20), and CD15, thus suggesting bladder metastasis from lung adenocarcinoma.

## Conclusions

Secondary cancers to the bladder are rare. The majority of them result from direct extension of tumours from surrounding organs, such as prostate, colorectal, and cervical carcinomas; the minority are metastasis originating from lymphoma/leukemias or, less frequently, from solid tumours such as breast, lung and skin primaries.

Differentiating secondary adenocarcinoma lesions from a primary bladder adenocarcinoma can be difficult. In patients without another known primary adenocarcinoma, the possibility of bladder cancer being due to spread of a contiguous-site malignancy should first be sought, but imaging studies of the chest, abdomen, pelvis and bones are also warranted not only for staging but also to rule out the presence of another possible primary malignancy elsewhere. Conversely, in patients known to harbour a distant site primary adenocarcinoma, the differential diagnosis between primary and metastatic bladder adenocarcinoma relies on endoscopic appearance [3] and pathologic evaluation.

In the few previously reported cases, the finding of an intact epithelium overlying the bladder tumour was considered suggestive of a secondary lesion [3]. Our case is the first jn which the endoscopic appearance was strongly consistent with primary bladder cancer. Such a finding, together with the 13-year history of self clean intermittent catheterization, further complicated the differential diagnosis between metastatic lung adenocarcinoma and primary bladder adenocarcinoma. The differential diagnosis, therefore, had to rely only on a thorough pathologic evaluation.

In the reported case, we elected to use to the TTF-1, CK7, CK20 immunohistochemical panel, which has recently been described as an effective means of differentiating lung adenocarcinoma from its metastases [6]. The use of a limited immunohistochemical panel including napsin A, a recently described highly sensitive marker for lung adenocarcinoma, and GATA3 and S100P, two novel markers of urothelial differentiation, has also been reported to be very useful in differentiating lung adenocarcinoma metastatic to the bladder from primary bladder adenocarcinoma [4]. In our experience, however, the TTF-1-CK7-CK20 panel proved to be easier to apply in routine clinical practice.

In conclusion, the differential diagnosis between secondary bladder cancers and non-transitional cell primary bladder cancers has major clinical relevance as primary bladder cancer is often curable with cystectomy and/or chemotherapy while bladder metastases from other primaries are managed with palliative chemotherapy and have a much worse outcome [7,8]. An endoscopic appearance consistent with primary bladder cancer, as in the reported case, may further complicate the diagnosis that should, consequently, be based on the patient’s history and, above all, on accurate pathologic evaluation and specific immunohistochemical staining.

## Consent

Written informed consent was obtained from the patient for publication of this case report and any accompanying images. A copy of the written consent is available for review by the Editor of this journal.

## Competing interests

The authors declare that they have no competing interests.

## Authors’ contributions

LC is responsible for conception and manuscript revision. FS performed the pathology evaluation and manuscript drafting. GDF conducted data analysis. PM undertook data acquisition. GL was involved in manuscript drafting. PB provided pathology supervision. GC provided supervision. All authors read and approved the final manuscript.
